# Rapid *S*-nitrosylation of actin by NO-generating donors and in inflammatory pain model mice

**DOI:** 10.1186/1744-8069-7-101

**Published:** 2011-12-22

**Authors:** Jingshan Lu, Tayo Katano, Daisuke Uta, Hidemasa Furue, Seiji Ito

**Affiliations:** 1 Department of Medical Chemistry, Kansai Medical University, Moriguchi, Japan; 2 Department of Information Physiology, National Institute for Physiological Sciences, Okazaki, Japan; 3 School of Life Science, The Graduate University for Advanced Studies, Okazaki, Japan

**Keywords:** dopamine release, F-actin, inflammatory pain, nitric oxide, PC12 cell, *S*-nitrosylation, spinal cord, *in vivo *patch-clamp recordings

## Abstract

**Background:**

*S*-Nitrosylation, the reversible post-translational modification of reactive cysteine residues in proteins, has emerged as an important mechanism by which NO acts as a signaling molecule. We recently demonstrated that actin is a major *S*-nitrosylated protein in the spinal cord and suggested that NO directly attenuates dopamine release from PC12 cells by causing the breakdown of F-actin. However, the occurrence of *S*-nitrosylation of actin remained unclarified in animal pain model. Kinetic analysis of *S*-nitrosylation of actin in the present study was made by using NO-generating donors. The biotin-switch assay and purification on streptavidin-agarose were employed for identification of *S*-nitrosylated actin.

**Results:**

Dopamine release from PC12 cells was markedly attenuated by NOR1 (*t*_1/2 _= 1.8 min) and much less by NOR3 (*t*_1/2 _= 30 min), but not by *S*-nitroso-glutathione, an endogenous NO donor. A membrane-permeable cGMP analogue could not substitute for NOR1 as a suppressor nor could inhibitors of soluble guanylate cyclase and cGMP-dependent protein kinase attenuate the suppression. *S*-Nitrosylated actin was detected by the biotin-switch assay at 5 min after the addition of NOR1. Consistent with the kinetic analysis, actin in the spinal cord was rapidly and maximally *S*-nitrosylated in an inflammatory pain model at 5 min after the injection of 2% formalin into the hind paws. *In vivo *patch-clamp recordings of the spinal dorsal horn, NOR3 showed an inhibitory action on inhibitory synaptic transmission in interneurons of the substantia gelatinosa.

**Conclusions:**

The present study demonstrates that rapid *S*-nitrosylation of actin occurred *in vitro *in the presence of exogenous NO-generating donors and *in vivo *in inflammatory pain model mice. Our data suggest that, in addition to the well-known cGMP-dependent protein kinase pathway, *S*-nitrosylation is involved in pain transmission via disinhibition of inhibitory neurons.

## Background

Abundant evidence has demonstrated that activation of the *N*-methyl-D-aspartate (NMDA) subtype of glutamate receptors and subsequent production of nitric oxide (NO) are key events in neurotransmission and synaptic plasticity in the central nervous system [1,2]. Different from many conventional neurotransmitters, NO, a reactive free-radical gas, simply diffuses from the nerve terminals into adjacent cells as anterograde and retrograde messengers and participates in numerous physiological and pathophysiological processes including nociception and pain hypersensitivity in the spinal cord [3-6]. We and others have demonstrated that NO contributes to the development and maintenance of hyperalgesia and allodynia in models of acute and chronic pain [7-10]. A rapid release of citrulline, a marker of NO synthesis, is observed in the spinal cord following a subcutaneous injection of formalin and is associated with a biphasic flinching behavior of the injected paw [11]. On the other hand, spinally administered NO donors depress ongoing impulse activity of dorsal horn neurons [12]; and inhibition of spinal NO synthase (NOS) leads to increased neuronal activity in the dorsal horn [13]. Thus the involvement of NO in pain is not consistent and is still controversial, probably due to differences in the experimental design and dose and nature of the agent employed [14,15]. The discrepancy may result from the existence of 2 signaling pathways of NO action. One is the classical pathway, where NO binds to the heme group of the soluble guanylyl cyclase (sGC) and activates it, leading to the generation of a second messenger, guanosine 3', 5'-cyclic monophosphate (cGMP), and then activation of cGMP-dependent protein kinase (PKG). The other is protein *S*-nitrosylation, i.e., the covalent attachment of a NO group to a reactive cysteine thiol, which has been recognized as a reversible post-translational modification [16,17].

Among methods for studying protein *S*-nitrosylation, the biotin-switch method has rapidly gained popularity because of the ease with which it can detect individual *S*-nitrosylated proteins in biological samples [18]. Over the past decade, the *S*-nitrosylation of more than 100 proteins, e.g., enzymes, transcription factors, ion channels, and structural proteins including NMDA receptors [19] and sGC [20], has directly been implicated in the regulation of cellular signaling pathways in intact cellular systems, based on data obtained by use of the biotin-switch method. We recently demonstrated that actin is a major *S*-nitrosylated protein in the mouse spinal cord, as evidenced by incubation of a spinal cord homogenate with *S*-nitroso-*N*-acetyl-DL-penicillamine (SNAP), an NO donor, and that it is also *S*-nitrosylated in PC12 cells [21]. NO decreases the amount of filamentous actin (F-actin), just like cytochalasin B, and attenuates the release of dopamine from PC12 cells. However, the relationship between actin *S*-nitrosylation with F-actin breakdown and inhibition of dopamine release remains unknown. To address this issue, we characterized the effect of NO donors on dopamine release from PC12 cells, using donors having controlled rates of NO generation, i.e., (±)-(*E*)-4-methyl-2-[(*E*)-hydroxyimino]-5-nitro-6-methoxy-3-hexenamide (NOR1), (±)-(*E*)-4-ethyl-2-[(*E*)-hydroxyimino]-5-nitro-3-hexenamide (NOR3), and SNAP, as well as *S*-nitroso-glutathione (GSNO), an endogenous NO donor [18]. Here we present that rapid *S*-nitrosylation of actin occurred *in vitro *in the presence of NOR1 and *in vivo *in the spinal cord of inflammatory pain model and correlated with the breakdown of F-actin and suppression of dopamine release from PC12 cells.

## Results

### Effect of NO donors on pituitary adenylate cyclase-activating polypeptide (PACAP)-stimulated dopamine release from PC12 cells

**Figure 1:**
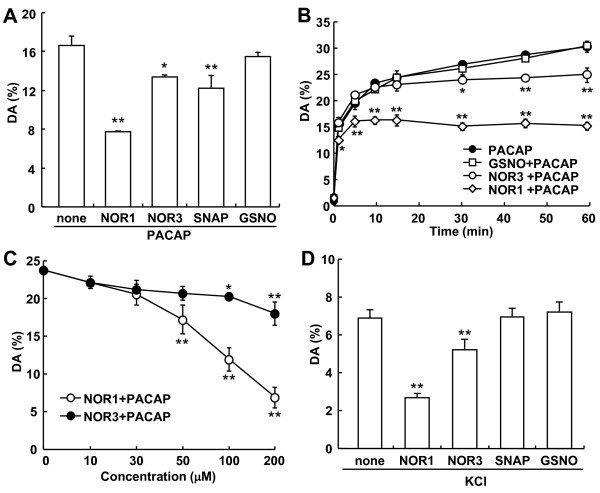
**Effect of NO donors on PACAP-stimulated dopamine release from PC 12 cells. A**. Inhibition of PACAP-stimulated dopamine release by NO donors. PC12 cells (4 × 10^5 ^cells/well) cultured on 24-well dishes were preincubated for 30 min with NOR1, NOR3, SNAP or GSNO (100 μM concentration of each) and then stimulated with 10 nM PACAP for 5 min in the presence of 10 μM imipramine, an inhibitor of catecholamine reuptake. Dopamine released into the medium and cellular dopamine were measured by HPLC as described in "Methods." *P < 0.05; **P < 0.01 *vs*. without NO donor. **B**. Time course of inhibition of PACAP-stimulated dopamine release by NO donors. PC12 cells were stimulated with 10 nM PACAP in the absence and presence of 100 μM NOR1, NOR3 or GSNO, and 10 μM imipramine for the indicated times. *P < 0.05; **P < 0.01 *vs*. PACAP alone. **C**. Concentration dependency of NOR1 and NOR3 for the inhibition of PACAP-stimulated dopamine release from PC12 cells. PC12 cells were stimulated for 10 min with 10 nM PACAP in the presence of various concentrations of NOR1 or NOR3 and 10 μM imipramine. Released dopamine (mean ± SD, n = 3) was expressed as a percentage of total dopamine (7.2 ± 0.8 ng/well) in PC12 cells. *P < 0.05; **P < 0.01 *vs*. without NO donor. **D**. Inhibition by NO donors of the KCl-stimulated dopamine release in PC12 cells. PC12 cells (4 × 10^5 ^cells/well) cultured on 24-well dishes were preincubated for 30 min with NOR1, NOR3, SNAP or GSNO (100 μM concentration of each) in the presence of 10 μM imipramine and then stimulated with 45 mM KCl for 5 min. Dopamine released into the medium (mean ± SD, n = 3) was measured as described above and expressed as a percentage of total dopamine in PC12 cells. **P < 0.01 *vs*. without NO donor.

PACAP is known to stimulate the release of dopamine from PC12 cells [22]. We recently demonstrated that the NO donor SNAP inhibits the PACAP-stimulated dopamine release from PC12 cells by causing the *S*-nitrosylation of actin [21]. To clarify how *S*-nitrosylation was involved in neurotransmitter release, we examined NO donors with different half-lives, NOR1 (*t*_1/2 _= 1.8 min), NOR3 (*t*_1/2 _= 30 min), SNAP (*t*_1/2 _= 6 h), and GSNO (*t*_1/2 _= 10 h), an endogenous NO donor, on the PACAP-stimulated dopamine release from PC12 cells. PC12 cells cultured on 24-well dishes were pre-incubated for 30 min with 100 μM NOR1, NOR3, SNAP or GSNO in the presence of 10 μM imipramine, an inhibitor of dopamine reuptake, and then stimulated for 5 min with 10 nM PACAP. Consistent with our recent findings that PACAP stimulated the release of dopamine, 16.6 ± 0.98% of the total dopamine, in PC12 cells and that SNAP attenuated it by 26.5%, NOR1 and NOR3 significantly reduced the PACAP-stimulated dopamine release by 53.2% and 19.4% (Figure 1A). GSNO had almost no effect on the release. When PC12 cells were stimulated with 10 nM PACAP in the presence of imipramine, dopamine release occurred in a biphasic manner: rapid release within 10 min and subsequent gradual release over 60 min (Figure 1B). When 10 nM PACAP and 100 μM NOR1 were simultaneously added to PC12 cells, NOR1 inhibited the first-phase release partially and the second-phase one completely. Unlike the inhibition by NOR1, the suppressive effect appeared only after 20-30 min with NOR3 and was not observed with GSNO (Figure 1B). The first 10-min release of dopamine was inhibited by NOR1 in a concentration-dependent manner, being 71.2% inhibition at 200 μM, whereas the suppressive effect by NOR3 was weak even at 200 μM (Figure 1C).

To clarify whether the suppressive effect of NO donors was specific to the PACAP-stimulated release, next we examined the effect of these NO donors on KCl-stimulated dopamine release under the same conditions as used for PACAP shown in Figure 1A. After a 30-min preincubation with NO donors in the presence of imipramine, PC12 cells were incubated for 5 min with 45 mM KCl. The dopamine released by 45 mM KCl was 6.91 ± 0.39% of the total cellular dopamine, smaller than that by 10 nM PACAP. The inhibition profile of KCl-stimulated release by the NO donors was similar to that for the PACAP-stimulated one; and the inhibition by NOR1 and NOR3 was 61.4% and 24.8%, respectively (Figure 1D). These results suggest that NO inhibited the dopamine release triggered by the 2 agents by a common mechanism.

### Inhibition of formation of F-actin by NO donor

**Figure 2:**
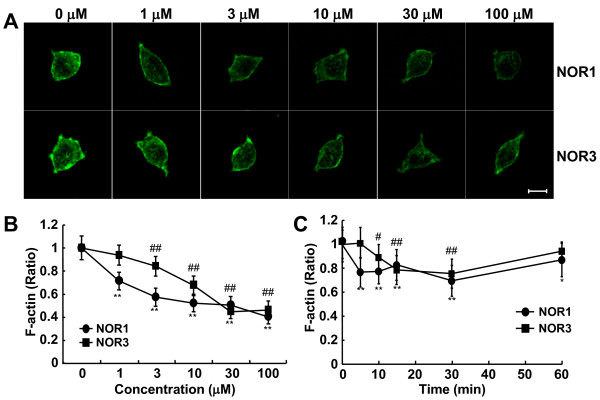
**Breakdown of F-actin in PC12 cells by NO donors. A**. Representative fluorescent images of F-actin in PC12 cells treated with various concentrations of NOR1 or NOR3. PC12 cells (3 × 10^4 ^cells/well) were incubated for 5 min with various concentrations of NOR1 or NOR3. Cells were fixed with 4% paraformaldehyde, and labeled with Alexa Fluor 488-phalloidin for F-actin. Bar = 5 μM. **B, C**. Concentration dependency (**B**) and time course (**C**) of F-actin breakdown by NOR1 or NOR3. Fluorescence intensity of more than 40 single cells/dish was quantified by ImageJ as described in "Methods." Data are the mean ± SEM of 3 independent experiments. **P < 0.01 *vs*. 0 μM NOR1; ^#^P < 0.05; ^##^P < 0.01 *vs*. 0 μM NOR3.

We demonstrated that actin is a major target protein for *S-*nitrosylation in PC12 cells and in the spinal cord [21]. To obtain more insight into the relationship between *S*-nitrosylated actin and attenuation of dopamine release by NO donors, we examined the effect of NO donors on the content and distribution of F-actin in PC12 cells. Since most of the dopamine release occurred within 10 min after the addition of PACAP, PC12 cells were treated for 5 min with various concentrations of NOR1 or NOR3; and the cells were then labeled with Alexa-phalloidin for F-actin. Whereas actin was broadly distributed in the cells, F-actin was mainly observed beneath the cell membrane (Figure 2A). Both NOR1 and NOR3 decreased the fluorescence intensity of F-actin (> 40 single cells/experiment, n = 3) in a concentration-dependent manner from 1 to 100 μM, being decreased maximally to 40-45% of the initial intensity. However, the concentration of NOR1 necessary for the half-maximal reduction was about 1 order lower than that of NOR3 (Figure 2B). To examine whether the reduction in F-actin by NO donors was recovered, we examined the fluorescence intensity for 60 min at 1 μM, at which concentration NOR1, but not NOR3, significantly decreased it at 5 min after the addition (Figure 2B). The fluorescence intensity of F-actin was significantly reduced to 76.8% at as early as 5 min after the addition of 1 μM NOR1, and the significant reduction continued up to 60 min (87.2% of the initial level, Figure 2C). On the other hand, NOR3 started to show its suppressive effect at 10 min and reached a level of reduction similar to that by NOR1 at 15 min. These results with NOR1 and NOR3 suggest that F-actin breakdown as well as the suppressive effect on dopamine release was dependent on NO produced from NO donors and that the breakdown continued for 60 min.

### No mediation of NO/cGMP/PKG signaling pathway in the inhibitory effect of NO on dopamine release

**Figure 3:**
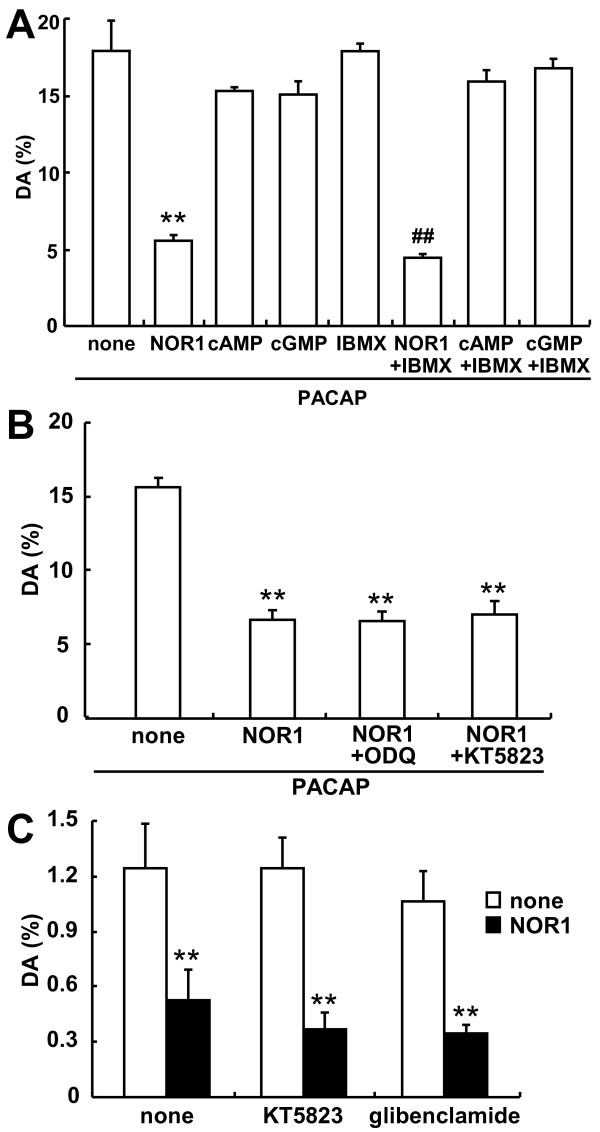
**No mediation of the cGMP/PKG pathway in inhibition of dopamine release by NOR1. A**. Effect of membrane-permeable cGMP and cAMP analogues on PACAP-stimulated dopamine release. PC12 cells (4 × 10^5 ^cells/well) cultured on 24-well dishes were stimulated for 10 min with 10 nM PACAP and 100 μM NOR1, 8-Br-cAMP or 8-Br-cGMP in the absence or presence of 100 μM IBMX. **P < 0.01 *vs*. none; ^##^P < 0.01 *vs*. IBMX. **B**. Effect of inhibitors of the cGMP/PKG pathway on PACAP-stimulated dopamine release by NOR1. PC12 cells were preincubated for 30 min with 100 μM NOR1 and 300 nM ODQ or 1 μM KT5823 in the presence of 10 μM imipramine. Then the cells were stimulated for 5 min by the addition of 10 nM PACAP. **P < 0.01 *vs*. PACAP alone (none). **C**. Effect of inhibitors of the cGMP/PKG pathway on basal dopamine release. PC12 cells were incubated for 5 min with 1 μM KT5823 or 1 μM glibenclamide without or with 100 μM NOR1. Dopamine was measured by HPLC, and dopamine released into the medium (mean ± SD, n = 3) was expressed as a percentage of total dopamine in PC12 cells, as described in "Methods." **P < 0.01 *vs*. none.

To clarify whether the suppressive effect of NOR1 on the dopamine release was mediated by the NO/cGMP/PKG signaling pathway, we examined this point by using agents related to the pathway. Compared to the marked reduction in dopamine release by 100 μM NOR1, membrane-permeable analogs of cGMP and cAMP, 8-bromo-cGMP (8-Br-cGMP) and 8-Br-cAMP, did not affect the PACAP-stimulated dopamine release, regardless of the presence of 100 μM 3-iso-butyl-1-methylxanthine (IBMX), a phosphodiesterase inhibitor (Figure 3A). Next we examined whether the suppressive effect by NOR1 could be attenuated by ODQ or KT5823, an inhibitor of sGC or PKG, respectively. When PC12 cells were preincubated for 30 min with ODQ (300 nM) or KT5823 (1 μM) in the presence of 100 μM NOR1 and 10 μM imipramine, neither ODQ nor KT5823 attenuated the suppressive effect of PACAP-stimulated dopamine release by NOR1 (Figure 3B). We further examined whether NOR1 reduced the basal release of dopamine. As with PACAP- and KCl-stimulated release, NOR1 decreased the basal release to 42.3%, which release was not affected by KT5823 or glibenclamide, an ATP-sensitive K^+ ^channel blocker [23] (Figure 3C). These results demonstrate that the suppressive effect of NOR1 was not mediated by the NO/cGMP/PKG signaling pathway.

### Dependency of actin S-nitrosylation by NO donors on NO generation

**Figure 4:**
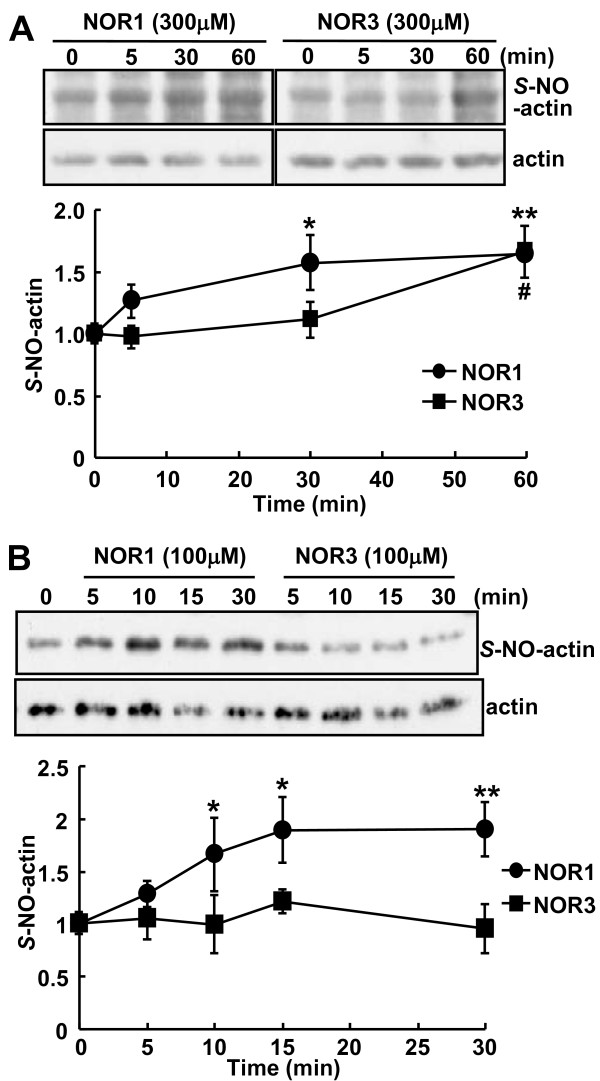
***S*-Nitrosylation of actin by NO donors. A**. Time courses of *S*-nitrosylation of actin in PC12 cells by NO donors. PC12 cells (7 × 10^5 ^cells/dish) cultured in 6-cm dishes were exposed to 300 μM NOR1 or NOR3 for the indicated times. The cell lysates in HEN buffer were subjected to the biotin-switch assay as described in "Methods." Samples were resolved by non-reducing SDS-PAGE and immunoblotted with anti-biotin and anti-actin antibodies for *S*-nitrosylated and total actin, respectively. Intensity of bands was quantified by using ImageJ. The extent of *S*-nitrosylated actin was normalized to total actin, and the ratio (mean ± SEM, n = 6) of *S*-nitrosylated actin to total actin at 0 min was taken as "1." *P < 0.05; **P < 0.01 *vs*. 0 min for NOR1; ^# ^P < 0.05 *vs*. 0 min for NOR3. **B**. Time courses of *S*-nitrosylation of actin by NO donors. Purified actin (5 μg) was incubated with 100 μM NO donor NOR1 or NOR3 for the indicated times and subjected to the biotin-switch assay. *P < 0.05; **P < 0.01 *vs*. 0 min for NOR1.

To clarify the difference in concentration dependency and time course of F-actin breakdown between NOR1 and NOR3, we measured *S-*nitrosylation of actin in PC12 cells by performing the biotin-switch assay. *S-*Nitrosylated actin was detected with anti-biotin antibody in NOR1-treated cells in a donor concentration-dependent manner (data not shown). When the band intensity of *S-*nitrosylated actin was normalized to that of total actin and the ratio without NO donor treatment was taken as 1, *S-*nitrosylated actin in PC12 cells rapidly increased 1.27-fold at 5 min after the addition of 300 μM NOR1 and 1.64-fold at 60 min. By the treatment of PC12 cells with 300 μM NOR3, *S-*nitrosylated actin was 1.66-fold increased at 60 min, comparable to that with NOR1 (1.64-fold, Figure 4A). Similar results on *S*-nitrosylation were obtained by the incubation of purified actin with 100 μM NOR1 or NOR3; the amount of *S-*nitrosylated actin rapidly increased 1.29- to 1.90-fold from 5 min to 30 min with NOR1, but it did not significantly increase until 30 min with NOR3 (Figure 4B). These results are consistent with the time course of suppression of the PACAP-stimulated dopamine release (Figure 1B) and F-actin breakdown (Figure 2C).

#### *In vivo S*-nitrosylation of actin in the spinal cord of inflammatory pain model

**Figure 5:**
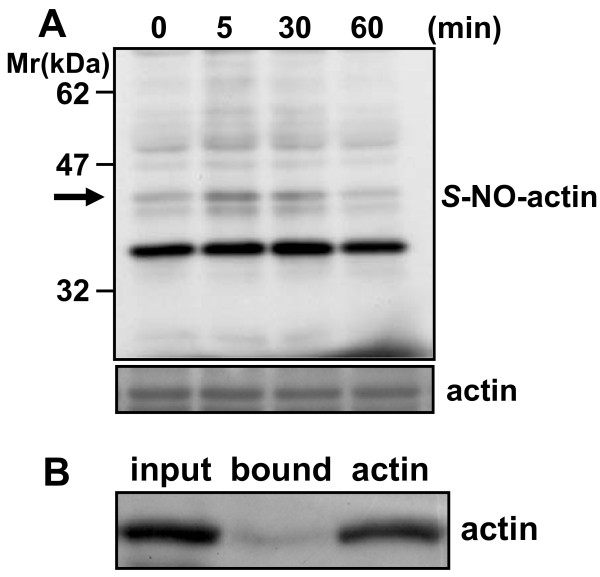
***In vivo S*-nitrosylation of actin in the spinal cord of inflammatory pain model. A**. Time course of *S*-nitrosylation of proteins in the spinal cord. The dorsal spinal cords at the L3-L5 levels were dissected at 0, 5, 30 or 60 min after injection of 2% formalin (5 μl) into the hindpaws, and homogenates prepared. The soluble fraction of the homogenates was subjected to the biotin-switch assay. *S*-Nitrosylated and total actin were detected by anti-biotin and anti-actin antibodies, respectively, as described under "Methods." An *arrow *indicates the position of *S*-nitrosylated actin (*S*-NO-actin). **B**. Purification of *S*-nitrosylated actin on streptavidin-agarose. After the soluble fraction prepared from the dorsal spinal cord at 5 min after formalin injection was subjected to the biotin-switch method, *S*-nitrosylated proteins were purified on streptavidin-agarose. The eluate was resolved on 10% SDS-PAGE, and *S*-nitrosylated actin was detected with anti-actin antibody. Authentic actin (1 μg) was used as a positive control.

Next, we examined whether actin was *S*-nitrosylated *in vivo *in inflammatory pain models. The dorsal spinal cords at L3-L5 levels were dissected before and 5, 30, 60 min after 2% formalin injection; and homogenates of them were prepared. Then the soluble fraction of the homogenates was prepared by a 20-min centrifugation at 100,000 × g. The extent of *S*-nitrosylation of proteins including actin was the highest at 5 min and gradually decreased at 30 and 60 min (Figure 5A). To confirm the *S*-nitrosylation of actin *in vivo*, we purified biotinylated proteins from the soluble fraction of the dorsal spinal cord dissected at 5 min after formalin injection by using a streptavidin-agarose gel and selectively eluted them with SDS-sample buffer containing 2-mercaptoethanol. Actin was detected in the eluate by anti-actin antibody (Figure 5B). However, *S*-nitrosylated actin was not detected by the biotin-switch method in the other inflammatory pain models: 6 and 24 h after injection of carrageenan and complete Freund's adjuvant (CFA), respectively (data not shown). These results demonstrate that *S*-nitrosylation of actin occurred rapidly *in vivo *in the spinal cord of inflammatory pain model mice.

To confirm this, we tried to detect *S*-nitrosylated proteins by a more sensitive immunohistochemical method using anti-nitrosocysteine antibody. We examined whether the antibody could be used for immunostaining of *S*-nitrosylated proteins in PC12 cells before using the spinal cord. The immunoreactivity of nitrosocysteine was dense in the area close to the membrane and co-localized with actin in PC12 cells (Additional file 1 - Figure S1). The intensity of immunoreactivity of nitrosocysteine was significantly increased 1.28- and 1.21-fold by 5-min incubation of PC12 cells with NOR-1 and NOR-3, respectively. These results demonstrated that *S*-nitrosylated actin in PC12 cells was immunostained with anti-nitrosocysteine antibody.

**Figure 6:**
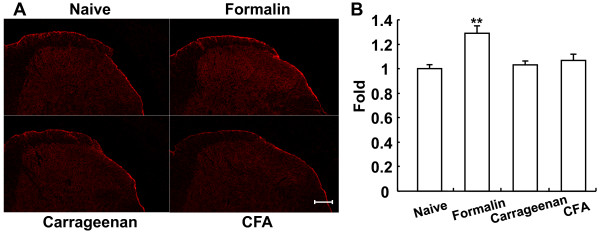
**Nitrosocysteine immunoreactivity in the spinal cord of inflammatory pain models. A**. Immunoreactivity of nitrosocysteine in the spinal cord. Lumbar transverse sections (20 μm) of spinal cords prepared from before and 5 min, 6 h, and 24 h after injection of formalin, carrageenan, and CFA, respectively, were fixed and stained with anti-nitrosocysteine antibody as described in "Method". Bar = 100 μm. **B**. Quantification of *S*-nitrosocysteine immunostaining. Fluorescence intensity of the spinal cord was quantified by ImageJ. The intensity of the spinal cord in inflammatory pain models was normalized to that of naive mice, and the intensity (mean ± SEM, n = 23-24) of naive mice was taken as "1." **P < 0.01.

Then we immunostained the spinal cord of inflammatory pain models with the anti-nitrosocysteine antibody. Transverse sections were prepared from the spinal cords before and 5 min, 6 h, and 24 h after injection of formalin, carrageenan and CFA, respectively. The immunoreactivity of nitrosocysteine was detected in the superficial layer of the spinal cord (Figure 6A). The intensity of immunoreactivity was significantly increased 1.29-fold by formalin, but not by carrageenan and CFA (Figure 6B). Consistent with the results of immunoblotting (Figure 5A), the immunohistochemical studies support rapid *S*-nitrosylation of proteins in the spinal cord *in vivo *in inflammatory pain model.

#### Inhibition of inhibitory postsynaptic current (IPSC) in the spinal cord by NOR3

**Figure 7:**
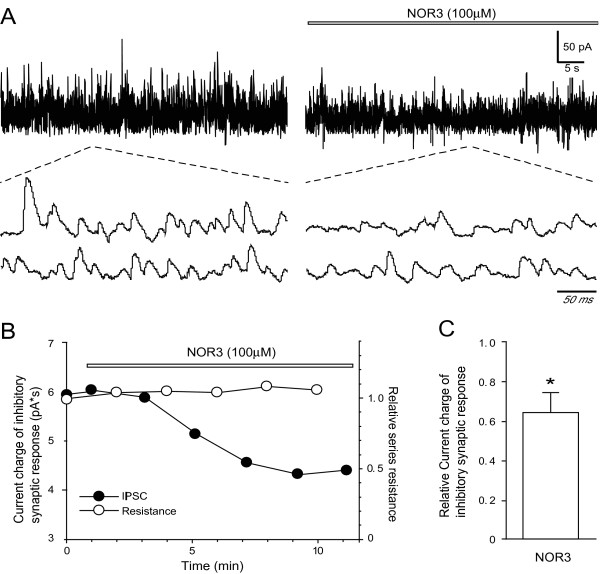
**Representative action of NOR3 on inhibitory synaptic transmission in the SG of the spinal dorsal horn *in vivo*. A**. Spontaneous IPSCs before and 8 min after (*left trace*) application of NOR3. Two lower consecutive traces of spontaneous IPSCs as shown in an expanded scale in time. **B**. Time course of the current charge amplitude of spontaneous IPSCs and relative series resistance of the recording under the action of NOR3. Data in **A **and **B **were obtained from the same neuron. **C**. Summary showing inhibitory action of NOR3 on inhibitory synaptic response. *P < 0.05 vs. control.

Finally, we examined NO action on inhibitory synaptic transmission in the substantia gelatinosa (SG) of the spinal superficial dorsal horn by using *in vivo *patch-clamp recording technique. Stable whole-cell recordings under voltage-clamp conditions could be obtained from 5 SG neurons for more than 20 min. SG neurons examined exhibited spontaneous IPSCs as previously reported [24]. When NOR3 (100 μM) with a longer half-life of 30 min was applied from the surface of the spinal cord for 8-10 min, the spontaneous inhibitory transmission was reduced as shown in Figure 7A. NOR3 gradually decreased the current charge of spontaneous IPSCs and reached a plateau ~8 min after the application (Figure 7B). The spontaneous inhibitory response (0.62 ± 0.06 of control, n = 5; P < 0.05) was significantly decreased by NOR3. These results suggest that NO has an inhibitory action on inhibitory synaptic transmission in the SG.

## Discussion

### Stimulation of glutamate release by the cGMP/PKG pathway and inhibition of dopamine release from PC12 cells by S-nitrosylation

NO, a reactive free-radical gas, simply diffuses from the nerve terminals into adjacent cells as anterograde and retrograde messengers and participates in nociception and pain hypersensitivity in the spinal cord [3,6]. NO binds to the heme iron of cGC and initiates the cGMP/PKG pathway. Based on previous reports indicating that paraformaldehyde-resistant NADPH-diaphorase (NADPH-d) activity is identical to neuronal NOS (nNOS) in the central nervous system [25,26], we demonstrated earlier that phosphorylation of NMDA receptor subunit NR2B at its Tyr 1472 and subsequent nNOS activation are involved in the maintenance of neuropathic pain [27-29]. Furthermore, to elucidate biochemical and molecular mechanisms for nNOS activation in the spinal cord, we established an *ex vivo *system for NADPH-d histochemistry [30], which enabled us to clarify whether NO itself regulated nNOS activity *in situ *by use of NO donors, NOR1, NOR3 and SNAP, with different half-lives of 1.8 min, 30 min, and 6 h, respectively [31]. Both NOR1 and NOR3 (100 μM), but not SNAP, enhanced the cGMP level 10- to 15-fold in isolated spinal cords in the presence of IBMX. Interestingly, whereas NOR1 slightly increased nNOS activity assessed by NADPH-d histochemistry at 100 μM, NOR3 markedly enhanced it at 10 and 100 μM [31]. We concluded that NO produced by NOR3 enhances nNOS activity mediated by glutamate release from synaptic terminals and activation of NMDA receptors via the cGMP/PKG pathway based on the following observations: (1) The NOR3-enhanced NADPH-d staining is completely inhibited by NMDA receptors and decreased in NMDA receptor NR2A knockout mice; (2) ODQ or KT5823 block the NOR3-enhanced NADPH-d staining; and (3) conversely, 8-Br-GMP intensely stimulated NADPH-d staining. In contrast to nNOS activation, the present study demonstrated that *S*-nitrosylation of proteins was rapid and that NOR1 was more effective in inhibiting dopamine release than NOR3 (Figure 1). Inhibition of dopamine release by NOR1 was not blocked by ODQ or KT5823 (Figure 3B). Furthermore, 8-Br-cGMP did not stimulate dopamine release from PC12 cells (Figure 3A). NOR1 inhibited the basal and 45 mM KCl-evoked dopamine release to a similar extent (Figures 1D and 3C). Taken together, these results demonstrate that the inhibition of dopamine release by NOR1 was mediated by *S*-nitrosylation, not by the cGMP/PKG pathway.

### Correlation of S-nitrosylation with inhibition of dopamine release and F-actin breakdown

PC12 cells are neurosecretory cells originally established from a rat pheochromocytoma [32]. PC12 cells synthesize and accumulate dopamine, but not noradrenaline or adrenaline, in sufficient quantity to be measured directly by HPLC. PACAP-stimulated dopamine release from PC12 cells was rapid and biphasic (Figure 1B). The inhibition of the release by NOR1 was completely blocked by 10 min. Since the inhibition by NOR3 was observed after 20 min, when most of the dopamine stimulated by PACAP had already been released, the extent of inhibition by NOR3 and SNAP was much lower as compared with that by NOR1 (Figure 1A). GSNO is a slow releaser of NO, as its decomposition rate is just approximately 5% per hour at room temperature. GSNO had no effect on the PACAP-stimulated dopamine release. In PC12 cells, secretory granules are concentrated close to the plasma membrane compared with other parts of the cytoplasm; and the compartmentalization of these granules is already pronounced under baseline conditions [33]. The low ability of PC12 cells to compensate for the release due both to poor synthesizing activity and poor storage machinery in PC12 cells may explain the time course of dopamine release from PC12 cells and the difference in the extent of inhibition among NO donors (Figure 1). Consistent with the distribution of secretory granules, F-actin was highly concentrated beneath the plasma membrane and formed the actin cortex in PC12 cells (Figure 2A). F-actin breakdown by 1 nM NOR1 was rapid and reached the maximum within 5 min, after which the breakdown continued for 30 min (Figure 2B). Increasing concentrations of NOR1 or NOR3 reduced the content of F-actin at most 60%. The kinetics of inhibition of dopamine release and F-actin breakdown in PC12 cells by NOR 1 and NOR3 was apparently well correlated with the *S*-nitrosylation of actin (cf. Figures 1, 2, and 4). The concentration-response curves for these effects were consistent between NOR1 and NOR3, suggesting that these rapid effects were dependent on the rate of NO generation from NO donors and were mediated by *S*-nitrosylation of actin and other proteins. However, whereas the decrease in fluorescence intensity of F-actin was detected at 1 μM NOR1, *S*-nitrosylated actin was detected at 100 μM NOR1 by immunoblotting. This difference may be attributed to the direct labeling of F-actin by phalloidin and sensitivity for detection. In addition, NO produced in the medium can easily and directly reach the F-actin network beneath the plasma membrane. On the other hand, *S*-nitrosylated actin was detected by the biotin-switch method as a small portion of the total actin in PC12 cells. Since *S*-nitrosylated actin was detected in the spinal cord 5 min after the formalin injection, NO produced in spinal neurons may have reached the actin network of adjacent neurons/glia and effectively *S*-nitrosylated the actin under the conditions of the *in vivo *milieu.

Actin filaments are important components of the cell cytoskeleton, where they are often involved in the process of exocytosis. Although the actin of rabbit skeletal muscle has 5 cysteine residues, at positions 10, 217, 257, 285, and 374, only Cys374 in the C-terminal region is exposed and important for polymerization [34]; and this residue is a decisive site of actin for *S*-nitrosylation [35]. We recently demonstrated that NO is an effective inhibitor of actin polymerization by causing *S*-nitrosylation [21]. The maximum reduction in dopamine release from PC12 cells was around 60% even after a 30-min pre-incubation of the PC12 cells with NOR1 before PACAP stimulation, suggesting that factors besides F-actin are also involved in the dopamine release from PC12 cells.

### Dichotomy of NO actions in the spinal cord and implication in pain

Interestingly, our previous and present studies using NO donors with different rates of NO generation showed that NO-enhanced *S*-nitrosylation of proteins was involved in the rapid response and that the NO-enhanced phosphorylation of proteins is involved in a long-lasting reaction in cells and tissues, and possibly *in vivo *in the spinal cord. In fact, whereas the *S*-nitrosylation of actin was rapid and maximal at 5 min after the formalin injection in the inflammatory pain model (Figure 5) and immunostaining of nitrosocysteine in the spinal cord was increased at 5 min after the formalin injection, but not 6 h after carrageenan injection and 24 h after CFA injection (Figure 6). On the other hand, phosphorylation of NR2B at its Tyr1472 and subsequent nNOS activation were observed 7 days after the operation in a neuropathic pain model [28,29]. Using a yeast two-hybrid screening, cysteine-rich protein 4 (CRP4) has been recently identified as a new effector downstream of PKG in the nociceptive system [36]. The dichotomy of NO actions was determined by the expression of proteins involving the NO/cGMP/PKG pathway. While nNOS is present in only 1-2% of lumbar dorsal root ganglion (DRG) neurons, nNOS-containing fibers and small interneurons are present in all layers of the spinal cord, especially in lamina II [37]. By contrast, PKGIα is expressed in small- or medium-diameter neurons of the lumbar dorsal root ganglia but is not present in spinal neurons and white matter tracks [37,38] and the majority of CRP4 mRNA-positive dorsal root ganglion neurons expressed PKG and peripherin [36]. Therefore, NO has been supposed to act as a retrograde messenger, i.e., to diffuse back to the presynaptic terminals of primary afferent fibers where it stimulates the release of glutamate via the cGMP/PKG pathway [3,31]. However, whereas CRP4-deficient mice showed no difference in nociceptive behaviors in acute pain and neuropathic pain, unexpectedly, they became hyperalgesic in inflammatory pain models as compared with wild-type mice [36]. In the present study, we demonstrated that actin was *S*-nitrosylated in the spinal cord *in vivo *in inflammatory pain model mice (Figure 5) and that NOR3 decreased inhibitory synaptic transmission in SG neurons of the dorsal horn by *in vivo *patch-clamp recordings (Figure 7), suggesting that attenuation of synaptic inhibition by *S*-nitrosylation may contribute to the manifestation of inflammatory pain. In this connection, sodium nitroprusside, another NO donor, inhibits ongoing impulse activity in 49% of all spinal neurons in laminas I and II [12], probably due to the lack of expression of PKG in these cells. We recently demonstrated that the nNOS inhibitor 7-nitroindole reduces the accumulation of activated microglia in the superficial layer of neuropathic pain model mice and blocks the migration of activated microglia *in vitro *[39]. Which types of cells possess *S*-nitrosylated proteins and whether disinhibition of inhibitory dorsal horn neurons by *S*-nitrosylation serves as pronociceptive in acute and chronic pain is an interesting topic in pain research.

## Conclusions

*S*-Nitrosylation has emerged as an important mechanism by which NO acts as a signaling molecule under pathophysiological conditions. The present study demonstrates that actin was rapidly *S*-nitrosylated *in vitro *by exogenous NO-generating donors and *in vivo *in the spinal cord of inflammatory pain model mice. NO showed an inhibitory action on inhibitory synaptic transmission in the spinal dorsal horn. These findings implicate *S*-nitrosylation in pain transmission via disinhibition of inhibitory neurons.

## Methods

### Materials

SNAP, ODQ, KT5823, and λ-carrageenan were obtained from Wako Pure Chemical (Osaka, Japan). NOR1 and NOR3 were purchased from Dojindo (Kumamoto, Japan). GSNO, *S*-methyl methanethiosulfonate (MMTS), imipramine hydrochloride, dopamine, 8-Br-cAMP, 8-Br-cGMP, IBMX, glibenclamide, actin, and CFA were purchased from Sigma-Aldrich (St. Louis, MO, USA). PACAP and *N*-[6-(biotinamido)hexyl]-3'-(2'-pyridyldithio)propionamide (biotin-HPDP) were supplied by Peptide Institute (Osaka, Japan) and Pierce Chemical (Rockford, IL, USA), respectively. Other chemicals were of reagent grade.

### Measurement of dopamine release from PC12 cells

Pheochromocytoma cell line PC12 cells were maintained in Dulbecco's modified Eagle medium supplemented with 5% fetal calf serum, 10% horse serum, and 50 U/ml penicillin and kept in a humidified environment of 95% air and 5% CO_2 _at 37°C. After PC12 cells had been seeded in 24-well plates (4 × 10^5 ^cells/well), they were cultured for 2 days and then preincubated for 30 min, if necessary, in 190 μl (or 180 μl) of HEPES buffer (in mM: NaCl 140, KCl 5, CaCl_2 _2, MgCl_2 _1.2, glucose 10, HEPES 10; pH 7.4); and then the appropriate agents (10 μl) were added once or twice to the medium. Incubation was carried out 37°C for the desired times in the absence or presence of 10 μM imipramine, an inhibitor of dopamine reuptake. After incubation, the culture medium in each well was harvested; and 3% perchloric acid in HEPES buffer was then added to each well for cell lysis. Culture media and cell lysates were adjusted to pH 4 by the addition of 1 M sodium acetate, and then the samples were centrifuged at 15,000 × g for 5 min. The supernatants of culture media and cell lysates were measured for dopamine released into the medium and cellular dopamine by using an HPLC column equipped with an Eicom electrochemical detector model 700 (Kyoto, Japan). HPLC was performed by using a reversed-phase C18 column (Eicom CA-50DS, 2.1 mm × 150 mm) with a phosphate-buffered mobile phase containing 20% methanol, 50 mg/L EDTA, and 0.5 mg/L sodium 1-octanesulfonate. The cellular dopamine content was 7.2 ± 0.8 ng/well; and basal and PACAP- and KCl-evoked release of dopamine into the culture medium was 1-1.5, 15-20, and 6-9% of cellular dopamine, respectively.

### S-Nitrosylation of actin and the biotin-switch method

*S*-Nitrosylated actin was detected by the biotin-switch method as described previously [16,19]. Briefly, 5 μg purified actin and 20 μg bovine serum albumin were incubated in the dark at room temperature with 100 μM NOR1 or NOR3 in HEN buffer consisting of 250 mM HEPES (pH 7.7), 1 mM EDTA, and 0.1 mM neocuproine. Then, the NO donor was removed from the reaction mixture by cold acetone precipitation; and the pellets were subsequently dissolved in HENS buffer containing 25 mM HEPES (pH 7.7), 0.1 mM EDTA, 0.01 mM neocuproine, and 1% sodium dodecyl sulfate (SDS), and blocked with fresh 4 mM MMTS for 20 min at 50°C. After the samples had been washed twice by acetone precipitation, the pellets were resuspended in HENS buffer and subjected to the biotin-switch assay, in which the sample was mixed with 1 mM ascorbic acid and 0.3 mM biotin-HPDP and kept for 1 h in the dark. Biotinylated actin was resolved by non-reducing SDS-polyacrylamide gel electrophoresis (PAGE), and detected by immunoblotting with peroxidase-conjugated anti-biotin antibody (1:1000; Sigma-Aldrich). The same membrane was stripped to detect total actin with anti-actin antibody (1:5000; BD Bioscience, San Jose, CA, USA) by using Enhanced Chemiluminescence (Amersham Biosciences, Piscataway, NJ, USA). The intensity of *S*-nitrosylated actin was quantified by using ImageJ software and normalized by that of total actin.

For *S*-nitrosylation of PC12 cells, the cells (7 × 10^5 ^cells/dish) were cultured on 6-cm dishes for 2 days, and the medium was replaced with serum-free medium 12 h prior to experiments. After treatment of the cells with 300 μM NOR1 or NOR3 for the indicated times, the cells were disrupted by sonication in HEN buffer; and the lysates were then subjected to the biotin-switch assay. The samples (30 μg protein/lane) were separated by non-reducing SDS-PAGE (10% gel) as described above.

### Preparation of inflammatory pain model and identification of S-nitrosylated actin

Male 5-week-old ddY mice were obtained from SLC (Hamamatsu, Japan). Mice were housed under conditions of a 12-h light: 12-h dark cycle, a constant temperature of 22 ± 2°C, and 60 ± 10% humidity. They received food and water *ad libitum*. Before and at 5, 30, and 60 min after injection of 5 μl of 2% formalin into the plantar surface of both hind paws, the mice were sacrificed; and then their dorsal spinal cords were removed at L3-L5 levels and homogenized in HEN buffer. The soluble fraction of the homogenate was obtained and subjected to the biotin-switch assay. The samples (20 μg protein/lane) were separated by non-reducing SDS-PAGE (10% gel) as described above.

For purification of *S*-nitrosylated proteins, the samples were prepared from dorsal spinal cords at 5 min after 2% formalin injection. After the biotin-switch assay, free biotin-HPDP was removed by use of an NP-5 column, and the eluate was incubated overnight at 4°C with 50 μl of a streptavidin-agarose slurry. The adsorbed *S*-nitrosylated proteins were eluted with SDS-sample buffer at room temperature for 20 min, and the eluate was resolved by 10% SDS-PAGE. Actin was detected by immunoblotting with anti-actin antibody. This study was conducted with the approval of the Animal Care Committee of Kansai Medical University and carried out in accordance with the ethical guideline of the Ethics Committee of the International Association for the Study of Pain.

### Fluorescence images for F-actin in PC12 cells

PC12 cells (3 × 10^4 ^cells/well) were plated on poly-L-lysine-coated glass-bottomed 35-mm dishes for 2 days. After the cells had been cultured overnight in serum-free medium, they were incubated without or with NOR1 or NOR3 in various concentrations for the desired times. Alteration of cellular F-actin was examined as reported previously [19]. Briefly, the cells were rinsed with phosphate-buffered saline, fixed with 4% paraformaldehyde in 0.12 M sodium phosphate buffer (pH 7.4) for 10 min, rinsed with phosphate-buffered saline 3 times, and blocked with 2% normal goat serum and 1% bovine serum albumin in phosphate-buffered saline for 30 min. F-actin was stained with Alexa Fluor 488-phalloidin (1:500, Invitrogen, Eugene, OR, USA) in phosphate-buffered saline for 2 h at room temperature. Digital images were captured by a Zeiss LSM510 laser-scanning confocal microscope (Oberkochen, Germany), and the fluorescence intensity was quantified by using ImageJ. The intensity of more than 40 single cells was quantified at each datum point, and 3 experiments were carried out for each analysis.

### Immunohistochemistry

Before and 5 min, 6 h and 24 h after injection of 2% formalin, 1% carrageenan, and 10 μl CFA (1 mg⁄ml mycobacterium in oil) into the plantar surface of both hind paws, mice were anesthetized and intracardially perfused with 50 ml of phosphate-buffered saline (PBS) followed by a fixative containing 4% paraformaldehyde in 0.1 M sodium phosphate buffer (pH 7.4). Lumbar spinal cords were removed and fixed in 4% paraformaldehyde and 0.2% glutaraldehyde in 0.1 M sodium phosphate buffer (pH 7.4) overnight at 4°C. After immersion in 30% (w/v) sucrose overnight, spinal cords were embedded in OCT medium and transverse sections (20-μm thickness) were cut by a cryostat. Then the sections were washed with 0.2% Triton X-100 in PBS, blocked with 2% normal goat serum and 1% bovine serum albumin in PBS for 1 h at room temperature. The sections were processed for immunohistochemistry with mouse anti-nitrosocysteine (1:2000, A. G. Scientific, San Diego, CA, USA) overnight at 4°C as primary antibody. The slides were washed three times in PBS and incubated with the Alexafluor 546 anti-mouse IgG (1:400; Invitrogen) as the secondary antibody for 1 h at room temperature. The immunofluorescence was visualized with a Zeiss confocal imaging system LSM510.

Sections (5-6/mouse) of a set of control and experimental spinal cords were concurrently immunostained and images were captured under the same conditions. The intensity of immunostaining was quantified by ImageJ.

### Elrectrophysiology

*In vivo *patch-clamp recordings from spinal dorsal horn neurons were carried out in normal 6 to 8-week-old rats essentially as described previously [40,41]. Briefly, under urethane anesthesia, a thoracolumbar laminectomy was performed, and then the animal was placed in a stereotaxic apparatus. The dura mater was removed, and the pia-arachnoid membrane was cut to make a window large enough to allow the patch electrode to enter the spinal cord. The surface of the exposed area of the spinal cord was irrigated with Krebs solution (in mM: NaCl 117, KCl 3.6, CaCl_2 _2.5, MgCl_2 _1.2, NaH_2_PO_4 _1.2, glucose 11, NaHCO_3 _25) equilibrated with 95% O_2_/5% CO_2_. NOR3 were added to the Krebs solution used for perfusion [41]. The patch pipettes were filled with a Cs solution (in mM: Cs_2_SO_4 _110, tetraethylammonium 5, CaCl_2 _0.5, MgCl_2 _2, EGTA 5, HEPES 5, ATP-Mg 5; pH 7.2). The tip resistance of the patch pipettes was 8-12 MΩ. Series resistance was assessed according to the response to a 5 mV hyperpolarizing step. This value was monitored during the recording session, and data were rejected if values changed by > 15%. The electrode was advanced into SG (lamina II) of the dorsal horn and then whole-cell voltage-clamp recording configurations were blindly performed from SG neurons. IPSCs were recorded under voltage-clamp conditions at a holding potential of 0 mV. The current charges of inhibitory synaptic responses were obtained from spontaneous IPSCs for 1 s.

### Statistics

Data for fluorescence images of F-actin were analyzed by one-way ANOVA, and statistical significance was further examined by Dunnett's test. Data for dopamine release, *S*-nitrosylation of actin, and electrophysiology were analyzed by paired Student's *t*-test. Data were presented as the mean ± SD or the mean ± SEM. P < 0.05 was considered statistically significant.

## Abbreviations

biotin-HPDP: *N*-[6-(biotinamido)hexyl]-3'-(2'-pyridyldithio)propionamide; 8-Br-cGMP: 8-bromo-cGMP; CFA: complete Freund's adjuvant; cGMP: guanosine 3', 5'-cyclic monophosphate; CRP4: cysteine-rich protein 4; F-actin: filamentous actin; GSNO: *S*-nitroso-glutathione; IPSC: inhibitory postsynaptic current; MMTS: *S*-methyl methanethiosulfonate; NADPH-d: NADPH-diaphorase; NMDA: *N*-methyl-D-aspartate; nNOS: neuronal NOS, NO: nitric oxide; NOR1: (±)-(E)-4-methyl-2-[(E)-hydroxyimino]-5-nitro-6-methoxy-3-hexenamide; NOR3: (±)-(E)-4-ethyl-2-[(E)-hydroxyimino]-5-nitro-3-hexenamide; NOS: NO synthase; ODQ: 1*H*-[1,2,4]oxadiazolo-[4,3-a]quinoxalin-1-one; PACAP: pituitary adenylate cyclase-activating polypeptide; PAGE: polyacrylamide gel electrophoresis; PKG: cGMP-dependent protein kinase; SDS: sodium dodecyl sulfate; SG: substantia gelatinosa; sGC: soluble guanylyl cyclase; SNAP: *S*-nitroso-*N*-acetyl-DL- penicillamine.

## Competing interests

The authors declare that they have no competing interests.

## Authors' contributions

JL was involved in data acquisition of all experiments. TK was involved in supervision of JL in all experiments. SI is a corresponding author and participated in the design of experiments and manuscript preparation. UD and FH were involved in *in vivo *patch-clamp recordings. All authors read and approved the final manuscript.

## Supplementary Material

Additional file 1 **Figure S1 - Validation of anti-nitrosocysteine antibody for immunostaining of *S*-nitrosylated proteins in PC12 cells**. **A-D**. Representative fluorescence images of immunostained PC12 cells with anti-nitrosocysteine and actin antibodies. After incubation for 5 min with 100 μM NOR1 (**A-C**), 100 μM NOR3 or 0.1% DMSO (**D**), PC12 cells (2 × 10^5 ^cells/well) were fixed with 4% paraformaldehyde and 0.2% glutaraldehyde, and labeled with anti-nitrosocysteine/Alexafluora 546-anti-mouse IgG (red) for *S*-nitrosylated protein (**A, D**), and anti-actin antibody/Alexafluora 488-anti-rabbit IgG (green) for total actin (**B**). Double labeling (**C**) was created by merging the images for nitrosocystein (**A**) and actin (**B)**. Immunohistochemistry in PC12 cells was carried out with anti-nitrosocysteine (1:2000, A.G. Scientific) and anti-actin (1:50 ZYMED, San Francisco, CA, USA) as described in "Methods". **E**. Negative control without primary anti-nitrosocysteine antibody. Bar = 10 μm. **F**. Quantification of immunoreactivities in PC12 cells. Fluorescence intensity was quantified by ImageJ. More than 60 cells were quantified at each datum point, and 3 experiments were carried out for each analysis. The nitrosocysteine immunoreactivity was normalized to total actin, and the ratio (mean ± SEM, n = 3) of DMSO was taken as "1". **P < 0.01. Click here for file
