# Supplementary figures and images for: Rapid S-nitrosylation of actin by NO-generating donors and in inflammatory pain model mice
Source: Mol Pain. 2011 Dec 22;7:101. doi: 10.1186/1744-8069-7-101 (PMC3295738; doi:10.1186/1744-8069-7-101)

## Slide 1
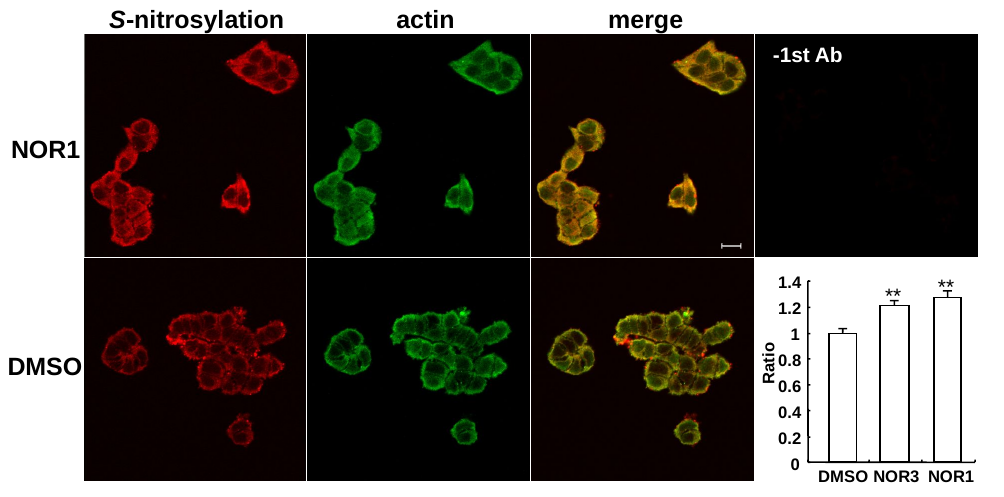

S-nitrosylation
actin
merge
-1st Ab
NOR1
**
1.4
**
1.2
1
DMSO
0.8
Ratio
0.6
0.4
0.2
0
DMSO
NOR3
NOR1

Supplement: Additional file 1 — Figure S1 - Validation of anti-nitrosocysteine antibody for immunostaining of S-nitrosylated proteins in PC12 cells. A-D. Representative fluorescence images of immunostained PC12 cells with anti-nitrosocysteine and actin antibodies. After incubation for 5 min with 100 μM NOR1 (A-C), 100 μM NOR3 or 0.1% DMSO (D), PC12 cells (2 × 10^5^ cells/well) were fixed with 4% paraformaldehyde and 0.2% glutaraldehyde, and labeled with anti-nitrosocysteine/Alexafluora 546-anti-mouse IgG (red) for S-nitrosylated protein (A, D), and anti-actin antibody/Alexafluora 488-anti-rabbit IgG (green) for total actin (B). Double labeling (C) was created by merging the images for nitrosocystein (A) and actin (B). Immunohistochemistry in PC12 cells was carried out with anti-nitrosocysteine (1:2000, A.G. Scientific) and anti-actin (1:50 ZYMED, San Francisco, CA, USA) as described in "Methods". E. Negative control without primary anti-nitrosocysteine antibody. Bar = 10 μm. F. Quantification of immunoreactivities in PC12 cells. Fluorescence intensity was quantified by ImageJ. More than 60 cells were quantified at each datum point, and 3 experiments were carried out for each analysis. The nitrosocysteine immunoreactivity was normalized to total actin, and the ratio (mean ± SEM, n = 3) of DMSO was taken as "1". **P < 0.01. [file 1744-8069-7-101-S1.PPT]
